# Educational strategies for general medicine education in accordance with the model core curriculum for medical education in Japan

**DOI:** 10.1002/jgf2.619

**Published:** 2023-04-01

**Authors:** Kiyoshi Shikino, Masaki Tago, Risa Hirata, Takashi Watari, Yosuke Sasaki, Hiromizu Takahashi, Taro Shimizu

**Affiliations:** ^1^ Department of General Medicine Chiba University Hospital Chiba Japan; ^2^ Department of General Medicine Saga University Hospital Saga Japan; ^3^ General Medicine Center Shimane University Hospital Shimane Japan; ^4^ Department of General Medicine and Emergency Care Toho University School of Medicine Tokyo Japan; ^5^ Department of General Medicine, Faculty of Medicine Juntendo University Tokyo Japan; ^6^ Department of Diagnostic and Generalist Medicine Dokkyo Medical University Tochigi Japan

## Abstract

In Japan, the Model Core Curriculum for Medical Education, which is the National Model COre CUrriculum for Undergraduate Medical Education, was first published in 2001 and has since been revised to meet the needs of an aged society and the global standardization of medical education. A new quality and ability “Generalism,” which is a comprehensive attitude toward patients, has been added to this revision of the model core curriculum. In this letter, we propose an educational strategy for generalism.

To the Editor

In Japan, the Model Core Curriculum for Medical Education, which is the National Model Core Curriculum for Undergraduate Medical Education, was first published in 2001 and has since been revised to meet the needs of an aged society and the global standardization of medical education.^1^ The model core curriculum was created by extracting core parts of the curriculum that should be commonly addressed by all universities when formulating their own curricula.^1^

In Japan, the proportion of patients with multiple comorbid medical conditions and complex social backgrounds is anticipated to escalate. Reflecting this rapid demographic change, training medical professionals who can respond to the drastic changes is socially important. For this reason, a new expertise quality and ability “Generalism,” which is a comprehensive attitude to approach toward patients, has been incorporated to this updated version of the model core curriculum.^1^ The objective of generalism is to “take a multi‐systemic view of the patient's problems and consider the patient's psychosocial background to provide comprehensive, flexible medical care that responds to the needs of the patient and is not limited to one's own specialty, supporting the achievement of individual and societal well‐being.”

The quality and ability set forth here and suitable for general medicine education. However, considering that only approximately 300 general medicine physicians are trained annually in Japan,^2^ an efficient and effective educational strategy is required to achieve the learning objectives. In this letter, we propose educational strategies for generalism.

On‐demand videos that can replace lectures should be made available and deployed as a shared resource throughout the country. In addition, support from academic organizations will be necessary to ensure high‐quality teaching materials. Video materials for documentaries and cine‐medications that allow students to visualize the actual site would also be useful. As a flipped classroom model, knowledge acquired in advance through video materials can be applied to early experiential training and clinical clerkships.^3^

It is also essential to share education resources (human resources, contents, and materials) in collaboration with diverse medical institutions outside the university rather than seeking resources only within the single university. Community hospitals and clinics are more likely to provide community‐oriented medical education and experience in the primary care field than university hospitals, which are higher level medical institutions. Furthermore, simply sending medical students to the community is not an effective form of education; the faculty members teaching there must also be able to provide high‐quality education.^4^ University hospitals, as conductors of the education system, are encouraged to provide educational support, conduct regular faculty development and train educators from extramural medical institutions.

Their primary role as conductors is to facilitate student reflection. Because vast amount of knowledge is learned in general medicine, the practice of self‐regulated learning is required for professional identity formation.^5^ Reflection with the supervising physicians on cases experienced is also crucial in this process. Significant event analysis may also be useful for reflection.

We believe that the future of medical education will change significantly as general medicine becomes more involved in the educational practices of the core curriculum.

## CONFLICT OF INTEREST STATEMENT

None.
